# Pancreatic Leiomyosarcoma With Multi-organ Metastases: A Rare Case

**DOI:** 10.7759/cureus.72520

**Published:** 2024-10-28

**Authors:** Safia Alnaqbi, Pawan Kumar, Abdulrahman Bin Sumaida, Nandan M Shanbhag, Amna Z Ahmad, Khalid Balaraj

**Affiliations:** 1 Oncology, Tawam Hospital, Al Ain, ARE; 2 Oncology/Radiation Oncology, Tawam Hospital, Al Ain, ARE; 3 Internal Medicine, College of Health Sciences, United Arab Emirates University, Al Ain, ARE; 4 Radiation Oncology/Palliative Care, Tawam Hospital, Al Ain, ARE; 5 Pathology, PureLab, Abu Dhabi, ARE

**Keywords:** advanced sarcoma management, aggressive malignancy, brain metastases, metastatic cancer, multidisciplinary oncology, palliative chemotherapy, palliative radiotherapy, pancreatic leiomyosarcoma, tumor pathophysiology

## Abstract

This case report describes the diagnosis, treatment, and progression of a 50-year-old male patient diagnosed with stage IV pancreatic leiomyosarcoma with metastases to the liver, lungs, and brain, representing the only case involving both the pancreas and brain documented in the hospital registry with a histopathological diagnosis of leiomyosarcoma. The patient initially presented with chronic abdominal pain, constipation, and weight loss, and subsequent CT scans revealed a large pancreatic mass with metastases to the liver, lungs, and brain. A biopsy confirmed the diagnosis of leiomyosarcoma, and the patient underwent eight cycles of palliative chemotherapy with cisplatin and docetaxel, along with palliative radiotherapy targeting the brain metastases. Despite these interventions, the disease progressed, resulting in the patient experiencing altered mental status, generalized weakness, and seizure-like activity, reflecting the impact of the brain metastases and overall disease progression. His management included treatment with dexamethasone, antiseizure medications, and supportive care, while discussions about palliative care options were conducted with the family. This rare case highlights the aggressive nature of pancreatic leiomyosarcoma, characterized by rapid progression and resistance to chemotherapy and radiotherapy, with the involvement of multiple organ systems demonstrating the challenges associated with managing advanced metastatic leiomyosarcoma.

## Introduction

Leiomyosarcoma is a rare, malignant smooth muscle tumor accounting for 5-10% of soft tissue sarcomas, typically affecting the uterus, gastrointestinal tract, or retroperitoneum. Pancreatic involvement of leiomyosarcoma is exceptionally rare, with few reported cases in the literature [1,2]. Even more uncommon is the occurrence of brain metastases in patients with leiomyosarcoma, as this tumor primarily metastasizes to the liver and lungs [3,4]. This case report describes the clinical course of a 50-year-old male diagnosed with stage IV pancreatic leiomyosarcoma with metastases to the liver, lungs, and brain, which represents a unique presentation in the hospital registry. This case highlights the aggressive nature of metastatic leiomyosarcoma and the challenges encountered in its management, despite the use of palliative chemotherapy and radiotherapy [5].

## Case presentation

**Figure 1:**
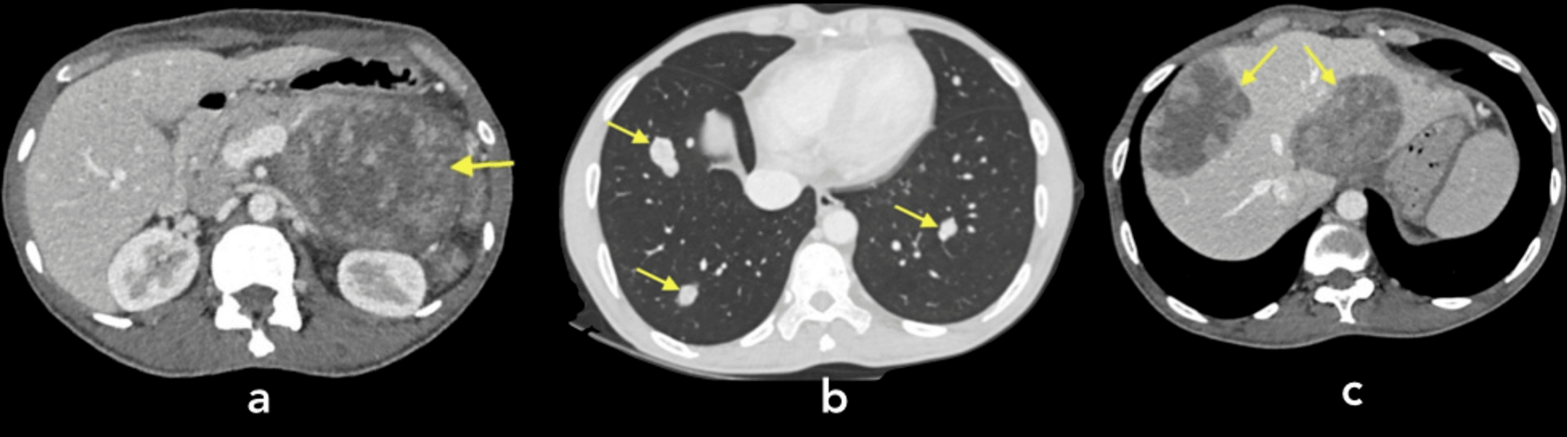
CT of the abdomen with contrast. Contrast-enhanced CT scan of the abdomen (a) showing a large heterogeneous mass in the pancreas, measuring approximately 10 × 9 × 12 cm, with evidence of necrotic foci (yellow arrow). The mass can be seen displacing adjacent structures, including the ileum, transverse, and descending colon, and causing a mild mass effect on the left kidney. Multiple metastatic nodules (yellow arrows) are also visible in both lungs (b). Metastatic lesions are present in the liver (c), with the largest lesion in segment II measuring 8 × 8 cm (yellow arrows).

A 50-year-old male presented to the hospital with complaints of chronic abdominal pain, constipation, and significant weight loss. His past medical history was unremarkable. In August 2022, CT imaging revealed a pancreatic mass measuring 10 × 9 × 12 cm, along with metastases in the lungs and liver (Figures 1a-1c).

**Figure 2:**
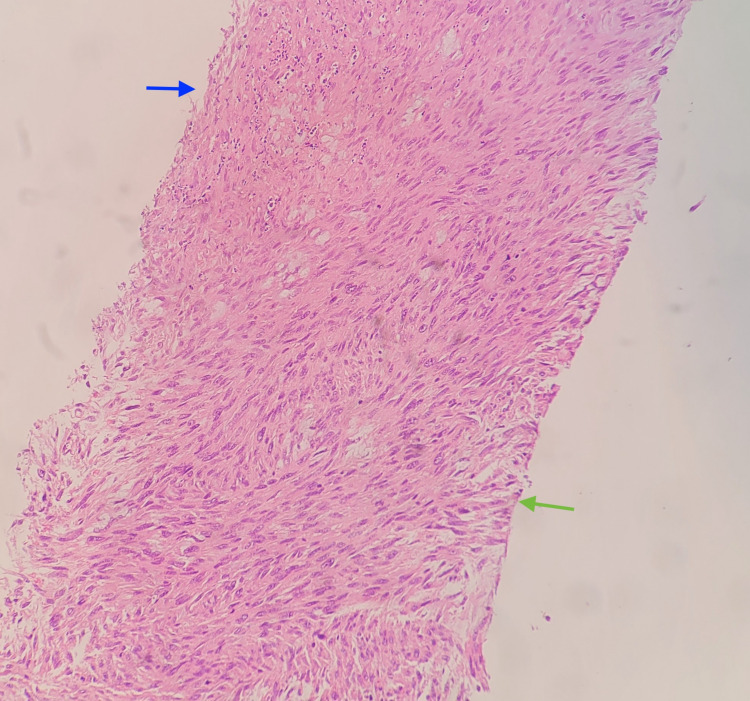
Pancreatic mass biopsy (hematoxylin and eosin (H&E) ×10). Histological examination with H&E staining reveals interlacing bundles of spindle cells with smooth muscle differentiation. There are hyperchromatic atypical nuclei (green arrow) with scattered mitosis and tumor cell necrosis (blue arrow).

A core needle biopsy of the pancreatic mass revealed a spindle cell tumor with significant mitotic activity (15 mitotic figures/10 HPF). The presence of tumor cell necrosis contributes to essential diagnostic criteria as well as poor survival (Figure 2).

**Figure 3:**
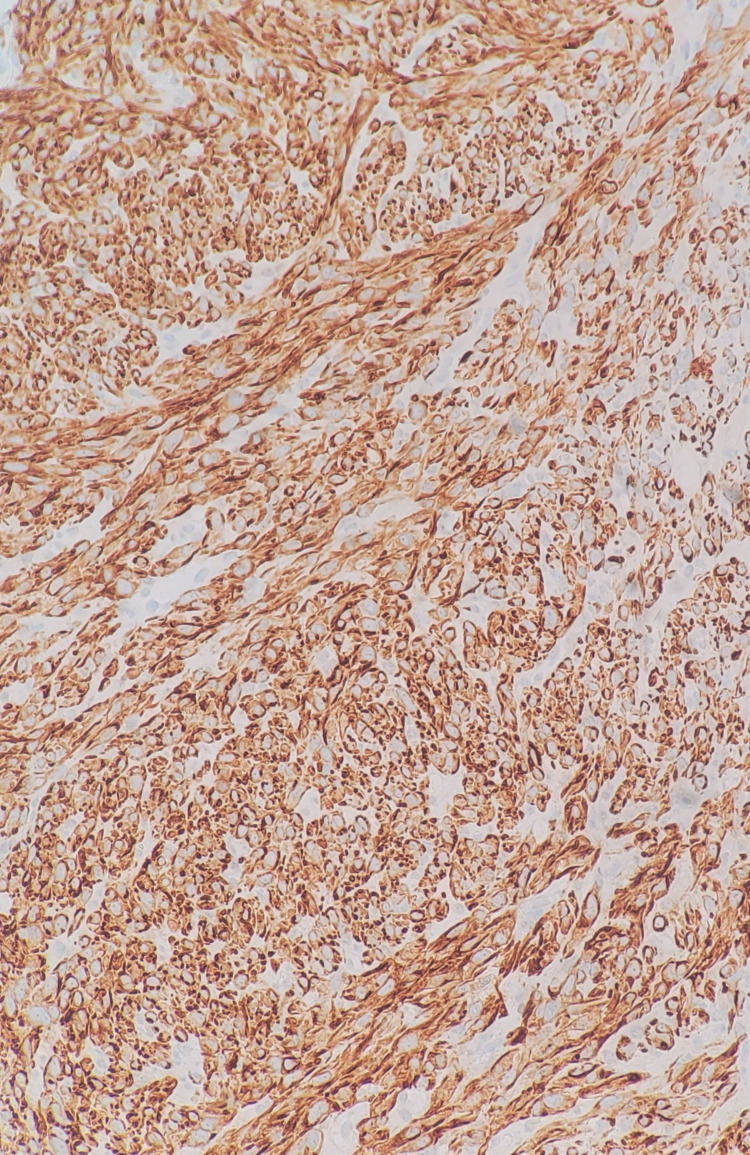
Immunohistochemical stain with caldesmon (×20). The cytoplasmic staining pattern is typical for leiomyosarcoma.

Immunohistochemistry confirmed leiomyosarcoma with positive staining for vimentin and caldesmon and a high proliferation index (Ki-67: 70%) (Figure 3).

**Figure 4:**
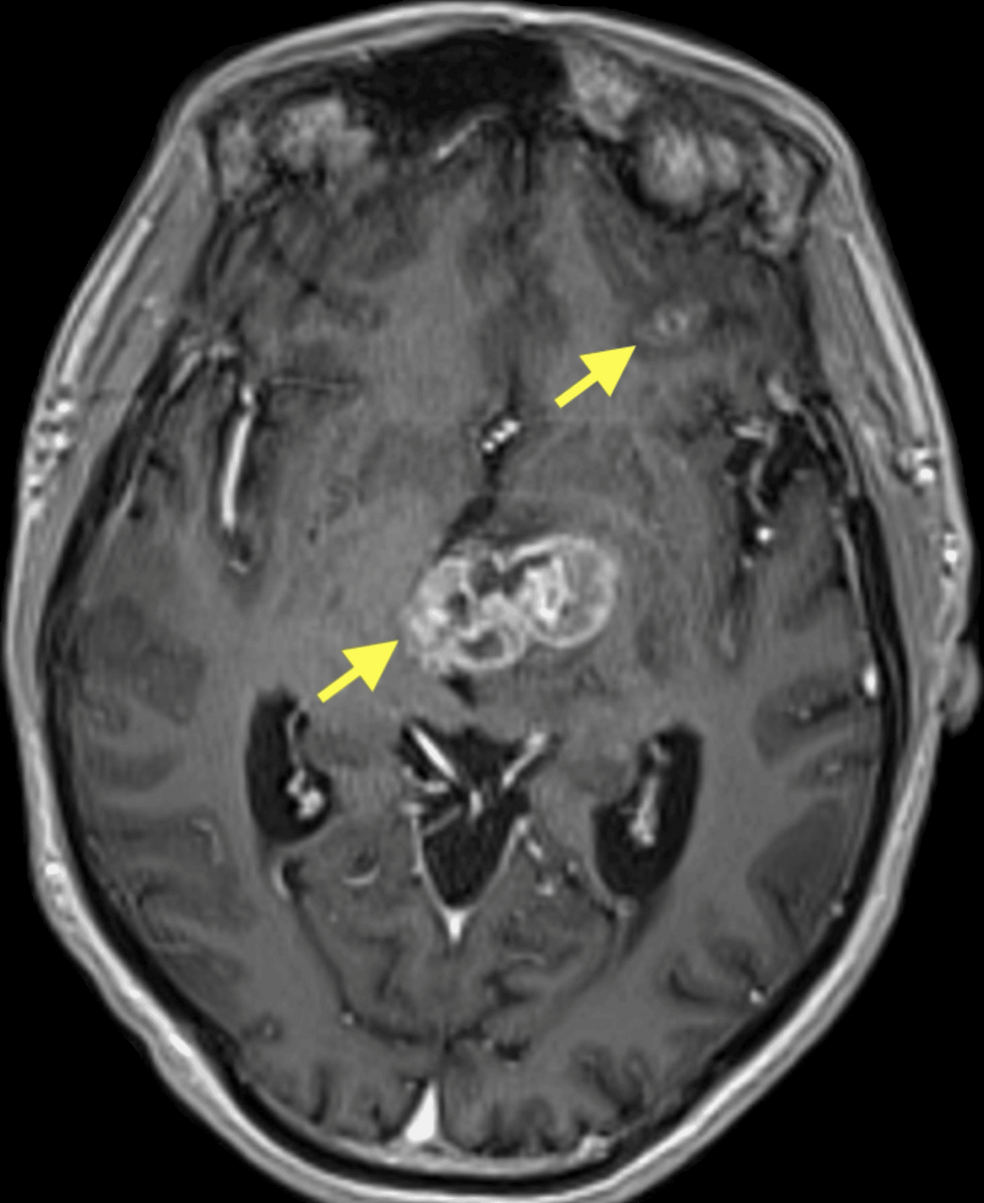
MRI of the brain T1 with contrast. MRI of the brain demonstrating the ring-enhancing multiple lesions in the left thalamus and the left frontal lobe (yellow arrows).

In September 2022, the patient began treatment with the first cycle of palliative chemotherapy consisting of cisplatin and docetaxel. During this time, he experienced intermittent episodes of dizziness and neurological symptoms, including left facial deviation and right-sided weakness. By October 2023, imaging confirmed brain metastasis with a significant hematoma in the left basal ganglia and thalamus, causing compression of the third ventricle. The left thalamic lesion measured about 3.9 × 3.6 × 2.1 cm, with low T1 signal intensity, mildly high T2 signal intensity, heterogenous post-contrast enhancement, and a mass effect over the midbrain and the left lateral ventricle and third ventricle with right-sided midline shift of about 9 mm (Figure 4). Three more sub-centimeter, ring-enhancing lesions were seen in the left frontal lobe and right temporal lobe.

**Figure 5:**
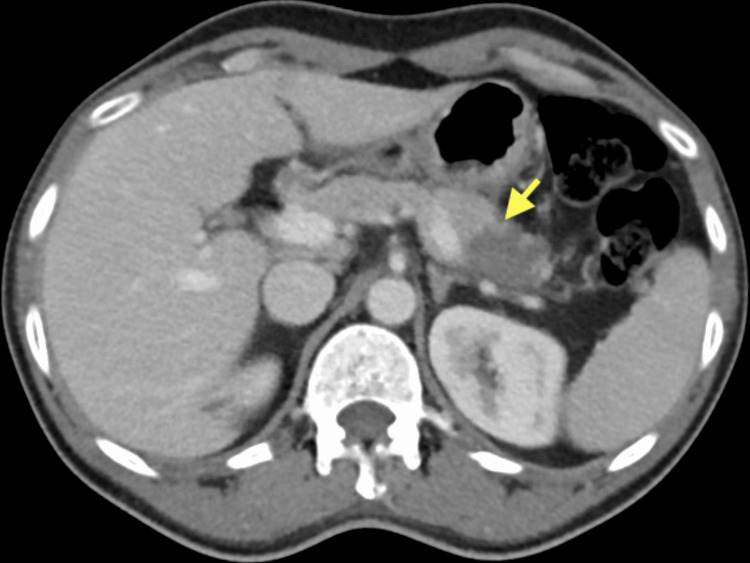
CT of the abdomen. CT scan demonstrating the interval reduction in the pancreatic lesion (yellow arrow).

The brain metastases were treated with palliative radiotherapy with a dose of 20 Gy in five fractions to the whole brain. In April 2023, reassessment imaging following eight cycles of palliative chemotherapy showed a reduction in the size of the pancreatic lesion (from 10 × 9 × 12 cm to 4.2 × 2.8 cm) and a reduction in lung lesions (Figure 5).

However, the progression of the disease in the brain continued, and the patient experienced ongoing neurological deterioration. By March 2024, the patient’s condition had worsened despite treatment, and he exhibited recurrent seizures and neurological decline, requiring dexamethasone and antiseizure medications for symptom management. In April 2024, the patient’s clinical status further deteriorated, with symptoms of altered mental status and aphasia. The patient was deemed unfit for any further cancer-directed treatment and referred to the palliative care unit for best supportive care.

Patient outcome

The patient succumbed to his illness 20 months after the initial diagnosis of stage IV pancreatic leiomyosarcoma with metastases to the liver, lungs, and brain. Despite aggressive palliative chemotherapy, radiotherapy, and supportive care, the disease continued to progress, ultimately affecting the patient’s neurological and overall functional status. He was admitted to the palliative care unit in his final months, where he received comprehensive symptom management, including dexamethasone for cerebral edema, antiseizure medications, and pain control. Under palliative care, the patient remained comfortable, and his symptoms were well managed, allowing for quality end-of-life care.

The cause of death was ultimately attributed to complications related to brain metastases, including progressive neurological decline, seizure activity, and respiratory failure, a common sequela in advanced metastatic disease. Despite the relentless progression of his illness, the patient passed away peacefully, surrounded by family, with his comfort prioritized through palliative care.

## Discussion

Leiomyosarcoma is a rare and aggressive soft tissue sarcoma arising from smooth muscle cells. While leiomyosarcoma primarily affects the uterus, retroperitoneum, and gastrointestinal tract, its occurrence in the pancreas is exceedingly rare, with few cases reported in the literature [1,2]. Due to its rarity, pancreatic leiomyosarcoma represents a diagnostic and therapeutic challenge. The prevalence of metastatic leiomyosarcoma is low, and its propensity to spread to the brain, as seen in this case, is even rarer, making this report significant in understanding its progression and management [3,4].

Prevalence

Pancreatic leiomyosarcoma is a rare subset of smooth muscle sarcomas. Soft tissue sarcomas account for less than 1% of all adult malignancies, and leiomyosarcoma accounts for 5-10% of these cases [3]. Primary pancreatic leiomyosarcomas are even more infrequent, with only a handful of documented cases. The incidence of metastasis to the brain in leiomyosarcoma is notably uncommon, as these tumors generally metastasize to the liver, lungs, and peritoneum. Brain metastasis from leiomyosarcoma, though rare, carries a poor prognosis, contributing to significant morbidity and mortality [4].

Presentation: signs and symptoms

Patients with pancreatic leiomyosarcoma often present with non-specific symptoms, which leads to delays in diagnosis. The clinical presentation typically includes abdominal pain, weight loss, and, occasionally, jaundice when biliary obstruction occurs [5]. In this case, the patient presented with chronic abdominal pain, constipation, and significant weight loss, all of which are common symptoms of advanced pancreatic malignancies. The presence of metastases complicates the clinical picture further.

Leiomyosarcoma, when metastasized, can present with symptoms depending on the site of metastasis. In this case, metastases were found in the liver, lungs, and brain. Brain metastases may present with neurological symptoms such as headache, altered mental status, seizures, or focal neurological deficits [6]. The patient in this case experienced altered mental status, general weakness, and seizure-like activity, which are characteristic of brain involvement by metastatic disease. These findings highlight the aggressive and multisystemic nature of metastatic leiomyosarcoma.

Management

The management of leiomyosarcoma, especially when metastatic, is complex and challenging. Surgical resection is considered the treatment of choice for localized leiomyosarcoma; however, in cases of advanced disease with metastases, surgery often plays a limited role [7]. The treatment paradigm shifts toward systemic therapies such as chemotherapy and palliative care.

In this case, the patient was treated with eight cycles of palliative chemotherapy using cisplatin and docetaxel. Chemotherapy is commonly used for metastatic leiomyosarcoma, although the response rate is variable and often suboptimal [8]. The combination of cisplatin and docetaxel is frequently employed in soft tissue sarcomas, though long-term survival benefits remain limited in cases of widespread metastases.

Palliative radiotherapy was administered to manage brain metastases, which is a standard approach for controlling symptoms and improving the quality of life in patients with brain involvement [9]. Radiotherapy is particularly useful in controlling seizures and neurological symptoms, though it does not halt disease progression. Additionally, the use of corticosteroids such as dexamethasone is important in managing peritumoral edema associated with brain metastases. Antiseizure medications were also a critical component of this patient’s care, addressing seizure-like episodes resulting from brain involvement.

Despite aggressive treatment with chemotherapy and radiotherapy, the patient’s disease continued to progress, underscoring the aggressive nature of metastatic leiomyosarcoma and its resistance to conventional therapies. Palliative care and discussions about end-of-life care were essential in this case, focusing on symptom management and quality of life.

## Conclusions

This case highlights the challenges of managing pancreatic leiomyosarcoma with multiple metastases. The rarity of pancreatic leiomyosarcoma and the involvement of the brain make this case unique and emphasize the need for further research into novel therapeutic options for metastatic leiomyosarcoma. Although palliative chemotherapy and radiotherapy can provide symptomatic relief, the prognosis remains poor in cases with extensive metastasis, highlighting the aggressive nature of this malignancy and the limitations of current therapeutic approaches.
